# Binary Simplification as an Effective Tool in Metabolomics Data Analysis

**DOI:** 10.3390/metabo11110788

**Published:** 2021-11-18

**Authors:** Francisco Traquete, João Luz, Carlos Cordeiro, Marta Sousa Silva, António E. N. Ferreira

**Affiliations:** Laboratório de FTICR e Espectrometria de Massa Estrutural, MARE-Marine and Environmental Sciences Centre, Faculdade de Ciências, Universidade de Lisboa, 1749-016 Lisboa, Portugal; franciscomrvrt@hotmail.com (F.T.); joaomluz97@gmail.com (J.L.); cacordeiro@fc.ul.pt (C.C.); mfsilva@fc.ul.pt (M.S.S.)

**Keywords:** metabolomics, data treatment, data analysis, Fourier Transform Ion Cyclotron Resonance mass spectrometry, multivariate analysis

## Abstract

Metabolomics aims to perform a comprehensive identification and quantification of the small molecules present in a biological system. Due to metabolite diversity in concentration, structure, and chemical characteristics, the use of high-resolution methodologies, such as mass spectrometry (MS) or nuclear magnetic resonance (NMR), is required. In metabolomics data analysis, suitable data pre-processing, and pre-treatment procedures are fundamental, with subsequent steps aiming at highlighting the significant biological variation between samples over background noise. Traditional data analysis focuses primarily on the comparison of the features’ intensity values. However, intensity data are highly variable between experimental batches, instruments, and pre-processing methods or parameters. The aim of this work was to develop a new pre-treatment method for MS-based metabolomics data, in the context of sample profiling and discrimination, considering only the occurrence of spectral features, encoding feature presence as 1 and absence as 0. This “Binary Simplification” encoding (BinSim) was used to transform several benchmark datasets before the application of clustering and classification methods. The performance of these methods after the BinSim pre-treatment was consistently as good as and often better than after different combinations of traditional, intensity-based, pre-treatments. Binary Simplification is, therefore, a viable pre-treatment procedure that effectively simplifies metabolomics data-analysis pipelines.

## 1. Introduction

Untargeted metabolomics experiments are focused on obtaining a global picture of a system with the objectives of identifying and characterizing all its metabolites or identifying key features, characteristics, and trends in the data that can help define and discriminate the systems under study [1,2,3]. At an individual level, every biological system is unique and will have a unique metabolome. Therefore, even two genetically identical systems will have minor differences in their metabolome. This uninduced biological variation will lead to inherent variability in the data [3,4]. The metabolome is extremely sensitive to experimental manipulation and environmental factors (slight changes in pH or growth medium, stress, temperature, among others), leading to considerable changes in metabolite concentration [3]. The variability in the metabolome justifies the need for great care in the proper quenching of cellular metabolism and the efficient extraction of metabolites during sample preparation. Moreover, the inherent metabolite concentration variability requires adequate statistical analysis.

Given its higher resolution, sensitivity, accuracy, and dynamic range, mass spectrometry (MS) is one of the selected analytical methodologies in untargeted metabolomics, leading to the detection of hundreds to thousands of variables and generating very complex data, which require robust and scalable computational and statistical tools to treat and extract meaningful information from them. An MS-based metabolomics dataset usually has a high number of variables, *m*/*z* values, also known as “features”, in comparison with the number of samples. A characteristic of these datasets is that a lot of these variables can be highly correlated due to physiological reasons, for example, for belonging to the same metabolic pathways or due to the sheer number of variables detected—the curse of dimensionality—leading to model overfitting when analyzed [5,6]. The variability present in each feature comes from the induced biological variation (that is, the intended variation to observe and analyze in the experiment) and the previously mentioned uninduced variation, which also encompasses all the technical variation due to either sample preparation or instrumental read-out variability. This variability can lead to large fluctuations in signal intensities not solely attributable to the induced biological variation. The different variables in a dataset can also be found within a large range of absolute magnitudes. Many multivariate statistical methods may give more weight to higher magnitude features, with larger absolute changes in concentration rather than lower concentration metabolites, which can be counteracted by proper data scaling. However, the biological importance of a metabolite may not depend exclusively on its concentration. For example, signal molecules usually have very low concentrations and can be fundamental in characterizing the phenotypes under study. Moreover, metabolomics data are usually heteroscedastic (the variability/variance of its features is not constant), while many different statistical methods assume the data are homoscedastic [4].

Robust computational and statistical tools have been developed and applied to treat and extract information from metabolomics data [3,5]. However, many of the currently applied methods have been adapted to the metabolomics framework from previously established “omics”, especially transcriptomics and proteomics [1,7], and consequently, they are not perfectly tailored to metabolomics.

After metabolite analysis, metabolomics data must be pre-processed, resulting in a 2D data matrix with variables represented in one dimension and samples in the other. Mass-spectrometry raw data processing includes spectra deconvolution, correction of the baseline and noise filtering, peak detection, or peak picking, peak alignment, and gap filling (if needed). In cases where MS is coupled with liquid or gas chromatography, retention time correction can also be employed [8,9]. Correction of the baseline is a noise filtering procedure used to remove low-intensity artefacts (generated from instrumental or experimental noise) by estimating the baseline shape and subtracting it from the raw signal [10,11,12]. The peak detection step aims to identify and quantify all features (ions) in the spectra while trying to avoid false positives, using, for example, peak-based methods (detect ‘peak-like shapes’) or binning-based methods (split spectra into small *m*/*z* intervals), [10,13]. After peak alignment, missing value imputation (gap filling) is performed between the pre-processing and pre-treatment steps in the data-analysis pipeline. Missing values arise in metabolomics datasets after peak or spectral alignments when a feature is not detected in a sample but is present in another. The imputation consists of filling those gaps with a value compatible with the different kinds of downstream data analysis steps (which may not adequately account for the presence of missing values) while maintaining the overall structure of the data. Missing values can be classified as missed completely at random (MCAR), missed at random (MAR), or missed not at random (MNAR), defining the missing-value imputation strategy [14,15]. The choice of the imputation method has a considerable effect on the data matrix and on the results of downstream statistical steps. After pre-processing, the obtained dataset must follow a pre-treatment with the objective of highlighting relevant biological information within the dataset while reducing the effect of undesired variation due to measurement or technical errors and slight changes in temperature, batch, or operator variation [4]. Data pre-treatment includes normalization, transformation, and centering and scaling. Normalization has the objective of suppressing between-sample global variation by trying to eliminate systematic bias related to sample dilution [8,11]. Transformations are a set of non-linear treatments whose main objective is to reduce heteroscedasticity and to make the data more symmetric (less skewed) [4,8]. Mean centering and scaling have the aim of “balancing” high and low intensity biologically important metabolites, and, to that end, they are often coupled together [4]. Each category contemplates multiple options of treatment and may also be applied in combination with each other [16], exponentially increasing the number of available options. Since the pre-treatments can considerably alter the results of the statistical analysis and constitute another layer of data variability, a thoughtful deliberation of the advantages and disadvantages of each treatment should be made, considering both the goal of the metabolomics study and the statistical analysis that will be performed downstream of the workflow [4,8].

Traditionally, data pre-treatments focus on the signal intensity values present in the dataset and are all intensity transformations, from the mathematical point of view. Despite maximizing the contribution of biologically important information contained in the intensities and, indirectly, in the metabolite abundances, these methods usually ignore (or diminish the relevance of) another important aspect of metabolomics data: the presence/absence of metabolites in a sample. This predominance of the signal intensity data in relation to the occurrence of spectral features is also supported by the traditional peak filtering/picking methods (exclude features that appear in a low number of samples) [14], followed by the imputation of missing values (which partially eliminates their information as non-detected metabolites). In general, the consideration of the occurrence of spectral features, as opposed to the intensities, is expected to be subjected to lower variability. Indeed, in a recent work, it was shown for a case study of untargeted metabolomics that the inter-laboratory compound annotation was more consistent than compound quantification [17].

**Figure 1:**
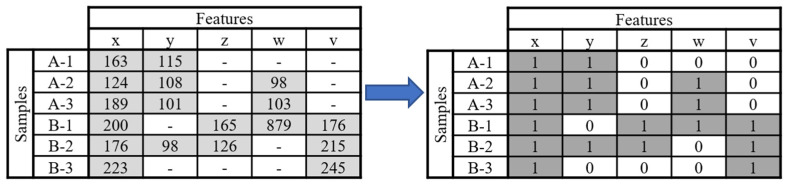
Outline of the Binary Simplification procedure: Numerical data matrices are transformed by encoding missing values with 0 and non-missing values with 1.

To make up for this gap in metabolomics and as an effort to make metabolomics data analysis more reproducible, we propose in this work a pre-treatment method based on the occurrence of features that forgoes the use of the more variable intensity data. In this method, named Binary Simplification (BinSim), feature occurrence in samples is encoded by 1 while feature absence is encoded by 0 in an already constructed data matrix (Figure 1).

This approach discards the variation in intensity data between different metabolomics experiments, even within the same batch, due to differences in sample preparation efficiency, dilution, or ionization efficiency. Lin et al. [17] reported the difficulty in having reproducible intensity and relative quantification results between two different laboratories that analyzed the same set of samples with the same protocol and different instruments, despite a good portion of the same metabolites being annotated in both analyses.

BinSim is a pre-treatment procedure that replaces most of the peak filtering, missing value imputation and pre-treatment methods of normalization, transformation and scaling used in the metabolomics data analysis pipelines and provides a single faster and simpler step, where no parameter-method choice or tuning needs to be made. Given its simplicity and aggressive truncation of signal intensities, one might wonder that information contained in data will be lost to an extent where the performance of downstream statistical methods will degrade. To demonstrate otherwise and establish BinSim as a viable procedure, we carried out an empirical comparison of the performance of clustering and classification methods applied to several experimental datasets transformed with BinSim or with six combinations of intensity-based transformations that can be considered representative of typical pre-treatment steps employed in metabolomics data-analysis pipelines. We conclude that BinSim does not degrade and often improves the performance of these methods.

## 2. Results

The BinSim pre-treatment was envisioned as a reliable and simpler alternative to more established intensity-based pre-treatments. It focuses on the presence or absence of features from the different samples instead of intensity-oriented pre-treatments. Furthermore, it was specifically created with ultra-high-resolution metabolomics data analysis in mind, where the occurrence of missing values is expected to be high. It consists of encoding intensity values as one for features present in a sample and zero for missing values, resulting in a binary data matrix comprised of 0 s and 1 s (as shown in Figure 1).

Several benchmark datasets were treated with seven different pre-treatment methods: BinSim and combinations of different pre-treatments widely used in metabolomics data analysis—missing-value imputation by either half the minimum value of the data matrix (1/2 min imputation) or Random Forest imputation (RF imputation) [18]; normalization, by reference feature or Probabilistic Quotient Normalization [19], depending on the dataset; generalized logarithmic transformation; and Pareto scaling [4,8]. The combinations were Pareto scaling only (P); normalization and Pareto scaling (NP); and normalization and glog transformation and Pareto scaling (NGP). Each of these was applied after either 1/2 min imputation or RF imputation, giving a total of seven pre-treatment combinations. The benchmark dataset matrices were constructed from the experimental data of three different sources: grapevine varieties, prefixed by GD; yeast strains, prefixed by YD; and a human dataset, referred to as HD. By performing different levels of variable selection related to spectral feature reproducibility, we were able to obtain eight datasets that display different basic characteristics such as the number of features and classes and cover a wide range of missing value percentages (Table 1).

Dataset *YD 6/15* was specifically created as an example of a scenario where the presence of missing values is low, which, a priori, is expected to reduce the performance of methods applied to BinSim treated data. Moreover, the datasets encompass different levels of class overlap as seen in Figure S1, with datasets *GDg2*+ and *GDc2*+ showing a high proximity of samples belonging to different classes in the PCA scores plots, *GDg2*− and *GDc2*− still problematic but with classes more spread out, *YD 2/15* and *YD 6/15* showing well-defined and separated classes, and the *GD types* dataset, used in the two-class classifiers, with classes also well separated. Class overlap is expected to dictate poor performance in both clustering and classification methods, since discrimination is expected to be hard. Furthermore, the differences in data characteristics also relate to instrumental performance since YD data were obtained in a more recent platform of Fourier Transform Ion Cyclotron Resonance (FT-ICR) instruments.

Therefore, the benchmark datasets represent several real-life scenarios of data quality levels in current high-resolution metabolomics.

The human dataset, also used in the two-class classifiers, represents a large LC-MS dataset with many samples per class but with comparatively low abundance of missing values and a high degree of class overlap.

To test the viability of the BinSim method in highlighting relevant information from metabolomics data, in the context of sample discrimination, the performance of two unsupervised, Hierarchical (HCA) and K-means Clustering, and two supervised statistical methods, Projection in Latent Structures Discriminant Analysis (PLS-DA) and Random Forest (RF), was compared. The ability to cluster together samples that are replicates was the criterion used to assess the performance of the unsupervised methods by defining “ground-truth” clusters, whereas predictive accuracy was the criterion used for classifiers. Furthermore, as it is a frequently performed step of metabolomics data analysis, feature importance evaluation in the classifier models was also analyzed. The goal was to investigate whether BinSim treated data offers a new perspective on the same original data (discrimination achieved by highlighting at a different set of features).

### 2.1. Unsupervised Statistical Analysis—Hierarchical and K-Means Clustering

**Figure 2:**
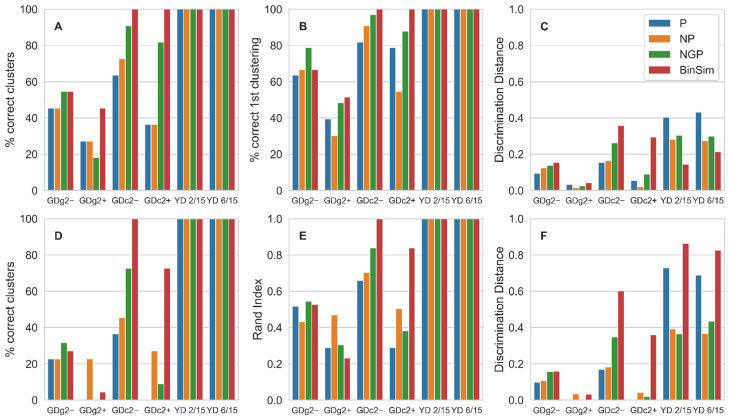
Effect of pre-treatments on clustering performance: (**A**) Correct clustering in HCA; (**B**) Correct first clustering in HCA; (**C**) Discrimination Distance in HCA; (**D**) Correct clustering in K-means clustering; (**E**) Rand Index in K-means Clustering; (**F**) Discrimination Distance in K-means clustering; Data pre-treatments: Pareto scaling only (P); normalization by reference feature and Pareto scaling (NP); normalization by reference feature and glog transformation and Pareto scaling (NGP); Binary Simplification only (BinSim). P, NP, and NGP were applied after either 1/2 min or RF missing-value imputation procedures. Except for BinSim, only the highest value of each measure between the two imputation procedures is plotted. The full results of clustering performance are shown in Tables S1 and S2.

The overall results of applying Hierarchical Clustering and K-means Clustering after each of the four pre-treatments are shown in Figure 2 and in Tables S1 and S2. As an example of the HCA, the resulting dendrograms for the dataset *GDc2*+ after BinSim and the three pre-treatments based on 1/2 min imputation are also shown in Figure S2 (for BinSim, the Jaccard dissimilarity was used as the binary distance metric).

The overall trend in these results is that the clustering methods applied to BinSim-treated data perform, in most cases, better than when applied to data subjected to the other pre-treatments. Considering the correct clustering percentage, which requires both completeness (all replicates cluster together) and homogeneity (each cluster contains only replicates) both methods achieve higher values with BinSim, except in the case of K-means Clustering for the two *GDg2* datasets, which are characterized for having samples from different ground-truth clusters very close together and might be considered low quality datasets. In fact, for *GDg2*+, both clustering methods perform poorly regardless of the pre-treatment procedure, with the correct clustering percentage below 50% in all cases. Even for these datasets, BinSim is not the worst pre-treatment. It is worth noticing that BinSim was the only pre-treatment to allow 100% correct clustering for the two *GDc2* datasets using HCA and for *GDc2*− using K-means. The results on the correct clustering percentage are qualitatively the same in HCA and K-means, even with the slight difference in definition: For HCA, “correct clustering” was defined as all the samples in a replicate group clustering together before clustering with a sample from any other group, whereas in K-means, the number of clusters in the result is prescribed as the number of replicate groups, and the definition requires homogeneity and completeness in the clusters obtained when the algorithm finally converges. Given these definitions, it is expected that correct clustering values will be lower for K-means when compared to HCA and that is, in fact, observed.

As the correct clustering percentage might be considered to have course granularity, since its values can only be multiples of 100/number of clusters, the correct first clustering percentage for HCA and the Rand Index for K-means were also calculated as other measures of clustering performance with finer granularity. Looking at these measures, the same overall trend holds, with BinSim being the best or second best except for the Rand Index for the low-quality dataset *GDg2*+ and being the only pre-treatment to achieve close to top values for *GDc2* datasets.

It is also important to note that, using these measures of clustering accuracy, perfect clustering was obtained with the two YD datasets in all cases, which is consistent with the fact that these are high-reproducibility datasets with replicate groups well separated from each other. This indicates that, also for data of high quality, BinSim does not degrade clustering performance.

Another measure for comparison, the Discrimination Distance, was also calculated. This measure represents the robustness of clustering performance to the negative impact of noise as it is an average of the normalized distance thresholds that, if overcome by outlier samples, would result in clustering errors. Datasets treated with BinSim consistently result in the best or close to the best value of Discrimination Distances when the K-means algorithm is used. As for HCA, BinSim only leads to lower Discrimination Distances for the two high-quality YD datasets. This qualitative discrepancy between HCA and K-means datasets might be related to the fact that in HCA the Jaccard dissimilarity was used as the distance metric for the BinSim-treated data and Euclidean distance in the other pre-treatments, whereas K-means uses Euclidean distance regardless of the pre-treatment. This is because K-means uses the projection of the samples in the n-dimensional space with n equal to the number of features, and therefore, binary distance metrics cannot be used to cluster the samples. So, the distance metric used was Euclidean for all the different datasets, including those treated with BinSim. Another reason might stem from the fact that the Discrimination Distance is defined differently in both methods, given their operating characteristics.

It is worth noting that the correct clustering percentage and Discrimination Distance are very sensitive to outliers with the “correct clustering” definition used, since just one stray sample from a group can lead to that entire group being labelled as not “well clustered”. However, this is not a problem for the results obtained since each dataset used here has only three replicates. Hence, one sample being an outlier in the group corresponds to a hefty part of the group and should be considered. It would be remiss to not say that these methods are not suited to be directly applied to test the clustering efficiency for datasets with higher number of samples per group. In these cases, they should be adapted. On the other hand, the correct first cluster percentage should be resistant to outliers as is.

These results using ground-truth clusters defined by replicates mean that, at a first look, the data treated with BinSim retain as much information as the other treated datasets to discriminate between the different groups. However, for HCA, it is interesting to investigate if during the agglomerative procedure some higher-level clusters are found to be common among the four pre-treatments. The interpretation of results based on these higher-level clusters is one of the motivations for using HCA, and the BinSim treatment should not destroy these relationships contained in data. A quick inspection of the dendrograms resulting from the four *GD* datasets (Figure S2) seems to reveal at least the CS/RL and RU/REG/TRI higher-level clusters. These are found regardless of the pre-treatments, including BinSim.

**Figure 3:**
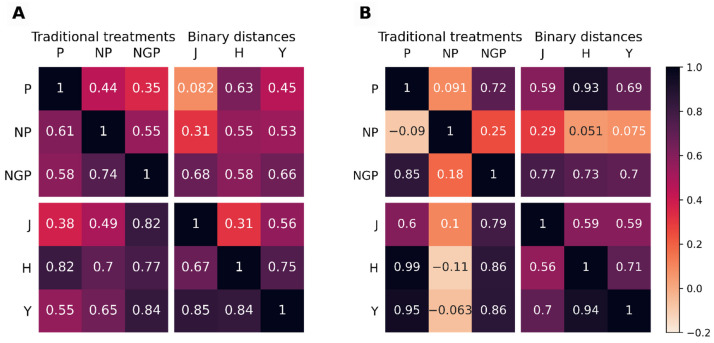
Correlations of HCA dendrograms using different pre-treatments or binary distances: Cophenetic correlations (lower triangular matrices) and Baker’s correlations (upper triangular matrices) for (**A**) dataset *GDg2*−; (**B**) dataset *GDg2+*. Data pre-treatments: Pareto scaling only (P); normalization by reference feature and Pareto scaling (NP); normalization by reference feature and glog transformation and Pareto scaling (NGP); Binary distances used in HCA after the BinSim pre-treatment: Jaccard distance (J), Hamming distance (H), and Yule distance (Y). For P, NP, and NGP, the Euclidean distance was used. A 1/2 min missing-value imputation was used in pre-treatments of P, NP, and NGP. Average linkage was used in all cases.

To have a more objective measure of the similarity between the dendrograms, the cophenetic correlation coefficient [20] and the Baker’s Gamma correlation coefficient [21] between all pairs of dendrograms were calculated. Figure 3 shows the heatmaps of such correlations for the two *GDg2* datasets, considered representative of lower quality data, with the Jaccard, Hamming, and Yule binary distance metrics used in HCA for BinSim-treated data while Euclidean distance was used in all three of the other pre-treatments.

Both correlation coefficients between the different dendrograms were, with few exceptions, higher than 0, and in fact, most of them indicate strong positive correlations. Furthermore, we could not establish the rule that intensity-oriented pre-treatments lead to clustering patterns quite different from those obtained from BinSim-based procedures: Correlations are not consistently higher between the three intensity-based pre-treatments than between these and BinSim with different distances and vice versa. For instance, NGP pre-treatment correlates higher with BinSim with all three distances in *GDg2*− than with P and NP. Conversely, BinSim with Jaccard has a lower correlation with BinSim with Hamming and Yule than with NGP in *GDg2*+. As another example, for *GDg2*+, the clustering resulting from HCA after NP has a low correlation with all other pre-treatments including BinSim with different distances. BinSim with the Hamming distance seemed to have slightly higher correlations with the P-, NP-, and NGP-treated datasets, but the dendrograms resulting from the three binary distance metrics were highly correlated (so any of the binary distance metrics led to very similar results) and were also usually positively correlated with the dendrograms treated with the other treatments (computed using the Euclidean distance). From these results, we reached the conclusion that data subjected to the Binary Simplification treatment revealed the same trends and information, as far as the metabolic similarity of the defined distinct groups are concerned, and that this can be extended to higher-level clusters in HCA.

### 2.2. Supervised Statistical Analysis—Random Forest and Projection in Latent Structures Discriminant Analysis Classifiers: Accuracy of Prediction

The next step was to compare the predictive performance of two types of classifiers, Projection in Latent Structures Discriminant Analysis (PLS-DA) and Random Forest (RF), considering the classes defined in the same way that they were defined for the comparison of the unsupervised methods. *GD types* was added to this benchmark, featuring a two-class problem targeting *Vitis vinifera* versus wild *Vitis* samples, based on *GDc2*− but with these different labels as targets. *HD* was similarly added to this benchmark, as another two-class problem with many samples per class but a rather low missing-value abundance and significant class overlap. These problems were created to benchmark BinSim’s effect on a common scenario in metabolomics data analysis: the use of two-class classifiers for discrimination and assignment of importance to features. As for the clustering methods, the seven pre-treatments were applied, and classification models were then built. Performance was evaluated by internal stratified cross-validation. The rationale for the inclusion of Random Forest was that the binary choices made during the operation of this algorithm would be able to extract the information present in a binary encoded dataset such as those resulting from the BinSim pre-treatment by choosing features where the two-levels would relate to class separation. This type of methods seems to be tailored to use BinSim treated data. On the other hand, PLS-DA is a dimension reduction classifier that was chosen due to its popularity in the metabolomics data analysis workflow [5,22].

**Figure 4:**
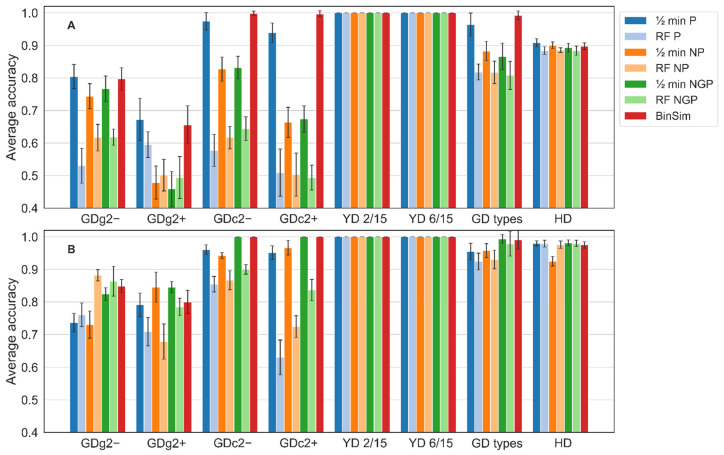
Influence of pre-treatment on the performance of classifiers: (**A**) Average accuracy of Random Forest; (**B**) Average accuracy of Projection in Latent Structures—Discriminant Analysis; Data pre-treatments: 1/2 min imputation and Pareto scaling (1/2 min P); RF imputation and Pareto scaling (RF P); 1/2 min imputation, normalization, and Pareto scaling (1/2 min NP); RF imputation, normalization, and Pareto scaling (RF NP); 1/2 min imputation, normalization, and glog transformation and Pareto scaling (1/2 min NGP); RF imputation and normalization and glog transformation and Pareto scaling (RF NGP); Binary Simplification (BinSim). Accuracy was estimated by 5-fold cross validation for *GD types* and *HD* and by 3-fold cross validation for all other examples and averaged over 20 cross-validation iterations, with error bars representing the standard deviation.

The results of the accuracy achieved by the two methods for data subjected to the seven pre-treatments are shown in Figure 4.

The results show that the BinSim pre-treatment does not compromise the performance of the classifiers developed for the eight problems included in this benchmark. For most examples, the classifiers perform better with BinSim than with the other pre-treatments, and in the cases where another pre-treatment results in better method accuracy, BinSim follows very closely, and the performance difference is only very slight. In absolute terms, the accuracy with BinSim-treated data follows the general trend of the clustering methods and leads to perfect classification in the case of the 5-class *YD* problems, close or equal to 1 in the 2-class *GD types* problem, and only leads to poor accuracy in the case of the two *GDg2 *examples, which are 11-class and difficult to discriminate problems, although still above 0.8 for *GDg2*−. It is worth noticing that the classifiers fitted on BinSim-treated data outperform the classifiers for the other treatments for the 11-class *GDc2* problems. With RF, it is the only treatment leading to perfect classification. Furthermore, regardless of the fundamental algorithmic differences, the results of PLS-DA are qualitatively consistent with the results of RF. The example of the large dataset *HD* suggests that BinSim does not degrade the performance of classifiers in large and complex datasets. Here, the type of classifier made a difference, with PLS-DA classifiers performing better than the corresponding RF classifiers. However, even for this example, classifiers for BinSim-treated data achieve an accuracy very close to the most accurate combination in the remaining six pre-treatments. For the *GD types* two-class dataset, ROC curves were also computed for the RF models built for BinSim and the three combinations based on 1/2 min imputation. These curves are shown in Figure S3, where the superiority of the RF model employed after the BinSim treatment is apparent.

The significance of the accuracy of the models was assessed by permutation tests, where the predictive accuracy with randomly permuted labels, estimated by cross-validation, was compared to the predictive accuracy of the corresponding non-permuted model (Table S3 and Figure S4). In all cases, the predictive accuracy distribution of permuted labels models was considerably below the non-permuted model accuracy (*p*-values < 0.02), which means that the classifiers’ accuracy resulted from significant information present in the data and not from random noise. This could also be concluded for the BinSim pre-treatment.

The main conclusion of this benchmark is that BinSim does not negatively impair classifier performance and often leads to its improvement. Despite the similar performances of the different models using the negative mode *GD* datasets, BinSim models seemed to perform slightly better than the other pre-treatments. It was also more consistent since it was the best or close to the best of all pre-treatments with RF, PLS-DA, HCA, and K-means in all the datasets studied. The information extracted from these datasets led to consistently good discrimination power of the numerous classes. These datasets work as a good proof-of-concept for the viability of BinSim with data difficult to discriminate. For the *YD* datasets, both RF and PLS-DA models had a perfect predictive accuracy (100%) in the discrimination of the five groups built on the models. This leads to the conclusion that the BinSim pre-treatment did not discard substantial and essential information by ignoring intensity data to hamper a perfect discrimination. Furthermore, comparing the results of *YD 2/15* with *YD 6/15*, the reduction of missing value abundance in the latter did not compromise such discrimination. Similarly, the lower abundance of missing values in *HD* dataset did not compromise the discrimination of the two classes.

### 2.3. Feature Importance in Classifiers

After establishing that the discrimination power of different statistical methods did not degrade with BinSim-treated data and that it was often improved, the next step was to investigate if BinSim was, as hypothesized, “looking at the information differently”, that is, if BinSim is giving more weight to information/features that are not as important in the results of the application of classifiers to data subjected to the other pre-treatments. Different “feature importance” metrics were used, specifically, the Gini Importance in RF models [23] and the Variable Importance in Projection (VIP) in PLS-DA models [24]. Importance scores were estimated with cross-validation and averaged for 20 iterations. The top 2% of features were retained. This fraction is usually much less than the fraction of features that is traditionally considered important when classifiers are used for feature importance assignment. For example, for the VIP metric, a usual cut-off threshold to keep a feature in feature selection is 1 [24]. In our benchmark, this cut-off would considerably increase the number of selected important features. Since the aim here was to compare the most essential features, only a smaller number of features was considered.

**Figure 5:**
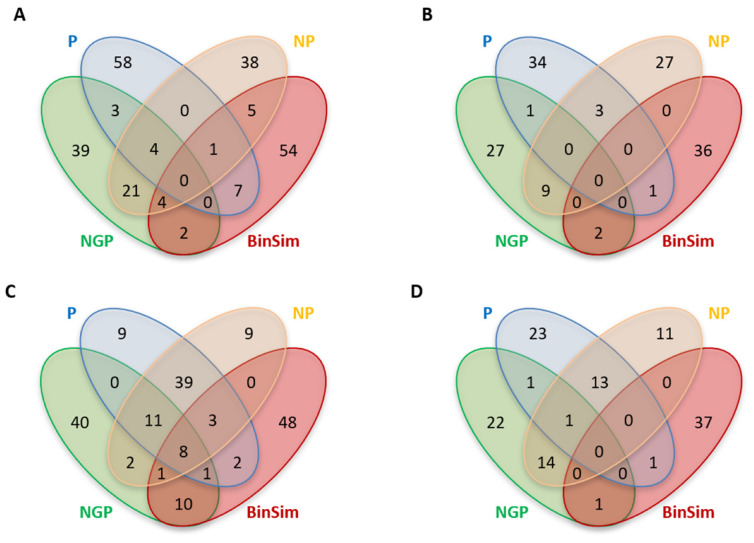
Overlap of the top 2% important features in classifiers fitted to data subjected to different pre-treatments: Random Forest models for (**A**) dataset *GDg2*− and (**B**) dataset *YD 2/15;* Projection in Latent Structures—Discriminant Analysis models for (**C**) dataset *GDg2*− and (**D**) dataset *YD 2/15;* Data pre-treatments: Pareto scaling only (P); normalization by reference feature and Pareto scaling (NP); normalization by reference feature and glog transformation and Pareto scaling (NGP); Binary simplification (BinSim). A 1/2 min missing-value imputation was used before pre-treatments P, NP, and NGP.

**Figure 6:**
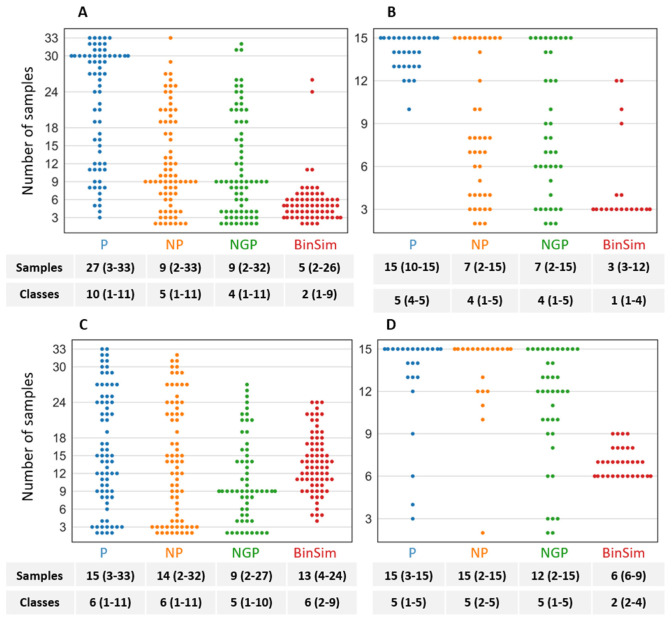
Distribution of sample occurrence of the top 2% important features in classifiers fitted to data subjected to different pre-treatments: Random Forest models for (**A**) dataset *GDg2*− and (**B**) dataset *YD 2/15;* Projection in Latent Structures—Discriminant Analysis models for (**C**) dataset *GDg2*− and (**D**) dataset *YD 2/15;* Data pre-treatments: Pareto scaling only (P); normalization by reference feature and Pareto scaling (NP); normalization by reference feature and glog transformation and Pareto scaling (NGP); Binary Simplification (BinSim). A 1/2 min missing-value imputation was used before pre-treatments P, NP, and NGP. Below each plot, the median and range of number of sample occurrences and median and range of the number of class occurrences are indicated.

Using the datasets *GDg2*− and *YD 2/15* as examples, and considering the top 2% important features, Figure 5 shows the overlap of important features between BinSim and the different pre-treatments based on 1/2 min missing-value imputation for the two types of classifiers. Figure 6 shows the distribution of important features according to their occurrence in samples considering the same pre-treatments, and the median and range of number of sample and class occurrences.

Considering the number of unique important features, BinSim-treated datasets have the most unique important features calculated from both classifier types, except for the case of the RF model applied to *GDg2*− with pre-treatment P where BinSim has the second highest number of unique features. Furthermore, the number of common features with the other treatments tends to be small.

Considering the distribution of the important features with the number of samples where they occur, both RF and PLS-DA models chose features for BinSim-treated datasets that appeared in a lower number of samples, except for the PLS-DA models applied to *GDg2*− after the NGP and NP treatments. These trends are also observed for the distribution by number of classes (the medians are indicated in Figure 6). This seems to point towards a conclusion that classifiers applied to BinSim-treated datasets tend to emphasize the information present in different features with different characteristics, when compared with the other pre-treatments. For RF models, it is clear that the important features have a bigger number of missing values and tend to be exclusive to one or very few classes. In class discrimination contexts, features exclusive to only one group act as potential “biomarkers”. These features are often discarded during traditional peak filtering for having too many missing values. For example, considering the top 2% important features for the RF model for the BinSim-treated dataset *GDg2*−, if a filter of 50% non-missing values was applied, then only two of these features would be retained. Only with more permissive filters would these features be carried over to further investigation: By allowing 80% missing values, 19% of these features would be retained, and allowing 90% missing values, 64% would be retained. When these features are kept, it is usually because feature filtering was conducted on an individual group basis. This kind of filtering would potentiate, in theory, the results of a method based on the occurrence of spectral features as opposed to intensity data, such as BinSim (highlights biomarker-like features). This was observed when this filtering was performed in the two *GDc2* datasets, leading to an increase in the performance of different methods for BinSim-treated datasets when compared to the *GDg2* datasets, where the reproducibility filter was global, considering all samples. It is interesting to note that the abundance of features that appear in very few classes in RF models of BinSim-treated data is not echoed in features that are present in almost every group except one or two. Therefore, this leads to the conclusion that there are more features that act like “biomarkers” than the opposite (are only absent in one or two classes).

The distribution of the feature occurrence by number of samples was very different in the RF and PLS-DA models. In the RF models, they mostly appeared in a very small number of samples ranging between three and six, that is, features that were exclusive to one class or sometimes two classes. This was more apparent in the *YD 2/15* dataset, where a significant number of important features occurred in only three samples, the number of samples in each class (Figure 6B). The choice of these features by the operation of the RF classifier are, most likely, due to the property that they identify and separate the samples belonging to one or sometimes two groups in one decision node in the ensemble of decision trees. On the other hand, in PLS-DA models, the important features tended to occur in approximately half of the samples for both datasets. For example, in *YD 2/15* (Figure 6D), most features occurred between 6 and 9 samples (with a higher number for occurrence in 6 samples) of the 15 total samples, that is, in 2 or 3 of the 5 different yeast strains. This difference from RF may be attributed to the fact that each component in PLS-DA is trying to maximize the global separation between all classes instead of prioritizing individual class separation. Using the example of *YD 2/15*, since features can only have two values (1 or 0), this happens when the contribution of a feature to a component separates approximately half of the classes between each other, that is, two classes from the other three; therefore, features that appear in two or three classes only (appear between six and nine samples) are prioritized.

Concerning the intensity-based pre-treatments, differences in the distributions of sample and class occurrences are clearly observed, not only between the two types of classifiers but also between the different pre-treatments. Even with RF, the information regarding the presence and absence of features becomes secondary, and class discrimination is often made by finding intensity patterns between the classes in features present in almost all samples. This is especially observed in P-treated datasets, where important features tend to occur in almost every sample and, consequently, every class, in contrast with the general trend of BinSim. This leads to the conclusion that the information regarding the presence and absence of features is being overlooked. On the other hand, the distribution for NGP is very spread out without a particular bias for samples that appear in a high or low number of samples. For NGP, there was a slightly higher occurrence of features that appear in multiples of three samples for the *YD 2/15* dataset. This higher occurrence can be explained by the fact that there may be some preference for features that only appear in some groups. This is an indication that there is still some consideration for the presence and absence of the features, which might be related to the fact that, among the intensity-based methods, the logarithmic transformation included in NGP decreases the difference between high-intensity values more than between smaller values, such as between imputed missing values and low intensity peaks, separating the “missing values” (absence of the features) more from the low-intensity features that count as present in the data. By attributing to these separations a slightly greater importance, when compared to other intensity-based pre-treatments, NGP acts closer to BinSim. In fact, in the Venn diagrams (Figure 5), the overlap between NGP and BinSim tends to be higher than with BinSim and the other two pre-treatments (despite still being small).

In summary, these results suggest that BinSim extensively changes classifiers’ important feature profiles. In BinSim-treated data, important features tend to work as indicators of groups as a direct result of the binary nature of data transformation. The differences from the features highlighted by classifiers after application of the other pre-treatments makes BinSim useful as a method that offers a different perspective on the information contained in metabolomics data without impairing the predictive performance of the classifiers.

## 3. Discussion

### 3.1. Advantages of BinSim

This empirical study suggests that BinSim is a viable alternative to traditional pre-treatment methods in metabolomics data analysis as it does not seem to impair the performance of downstream methods. As such, the main advantage of BinSim is clearly procedural simplification since it substitutes a multi-step pre-processing pipeline for a one-step data transformation. Apart from the gain in processing speed, the most important consequence of such substitution is that fewer choices about methods, options and parameters need to be made. BinSim works as a missing-values imputation, a normalization, and a scaling procedure out of the box: zero flags missing values, and variables are bounded by zero and one regardless of their former absolute magnitude. Global intensity biases that justify the need for normalization methods between samples are also averaged and thus attenuated by BinSim. It is worth noting that practice and benchmarking studies have shown that the choice of method for missing-value imputation, normalization, and scaling may have a significant impact on the results of data analysis in metabolomics and that there is not a universal choice in combinations of methods and parameters that works best in every study. This study also points out exactly that, by showing the inconsistency between the results of the six intensity-based pre-treatments applied to the same datasets.

The presence or absence of features can be very helpful in the discrimination of classes or groups especially for metabolites that are exclusive to one of the classes for a metabolomics study (and act as putative biomarkers in this context), or, conversely, when key features are absent from just one or two classes. However, this kind of information tends to be overshadowed by the intensity data in the usual metabolomics workflow due to the extensive peak filtering, missing value imputation, and the intensity-oriented nature of the traditional pre-treatments. Peak filtering tends to exclude features with higher amounts of missing values. Depending on the filtering method, those might include features exclusive to one or a few classes, overlooking the importance of these features in discrimination. The estimates of the loss of BinSim-RF important features for the example *GDg2*− shows that the use of common thresholds of non-missing-value abundance, say 50%, could result in the exclusion of most of those features, which are informative by their sample occurrence. For the application of BinSim, as shown in this study, the use of a filtering procedure related to missing-value abundance is not strictly necessary. The imputation of the missing values after filtering is an almost mandatory step for further statistical analysis since many statistical methods do not work with missing data. When these values are related to mistakes in the acquisition or processing of the data, that is, as MAR/MCAR values [14], their potential value for discrimination is lost. When they are MNAR values, usually resulting from metabolites that are absent or in very low concentrations [15], they are usually replaced by small values and partially retain their information and importance as these values are computed to express low concentration or absence. However, non-missing low intensity spectral features can be closer to (originally) missing values than to higher intensity features and may be treated in the same way as these by some statistical methods. This diminishes the importance of missing values in discrimination. With BinSim, MNAR values become informative as missing values and zeros in the encoding, fully contrasting with intensity features, even those with low concentration.

The good performance observed with BinSim-treated datasets might be a consequence of the big inherent variability of the intensity data of FT-ICR-MS that reduces the efficiency of the other treatments. With a lower sample/class ratio, trying to discern intensity patterns that reliably discriminate the different classes is challenging for classifiers and, therefore, more prone to errors, since an incidental pattern found in a very limited number of training samples might not be replicated in the corresponding test set, leading to misclassifications. We hypothesize that the BinSim’s robustness is higher (in comparison to other treatments) on low- to medium-sized datasets, where the amount of training samples to build a classifier is limited and relying on intensity patterns may be more prone to errors because of their variance. This could possibly be explored in future studies.

Another advantage of BinSim is that the results of the binary encoding are less dependent on instrumental settings and data analysis stages upstream pre-treatments. There are few operational settings that determine how a data point will be binarily encoded. One of them is the alignment of spectral features between the ensemble of samples, which constructs the data matrix, and is, in turn, dependent on instrumental measurement accuracy (*m*/*z* in MS-based metabolomics, chemical shifts in NMR-based metabolomics) and the tolerance prescribed for the deviations between spectral feature values between the different samples. However, this spectral alignment will influence the results of all pre-processing pipelines and will not specifically affect BinSim in any distinctive way. The signal-to-noise ratio is another instrumental parameter that will affect the results of BinSim since it will directly dictate whether a feature is a missing value by thresholding its intensity. In addition to affecting all other pre-treatments, the signal-to-noise ratio impact is not expected to be of great concern because it leads to relative, not absolute, thresholds. Since BinSim substitutes the normalization step, the overall intensity biases in samples, which are corrected by normalizations, could, in principle, cause differences in missing/non-missing value assignments of spectral features. However, constant signal-noise-ratio thresholds mitigate the effect of these biases since the baseline noise level variations tend to follow the general signal intensity variations of a sample. Therefore, it is expected that features be classified as missing/non-missing almost independently of overall intensity differences between samples.

A final advantage of BinSim is robustness to data leakage along the pre-processing pipelines. When BinSim is used to transform data, in each variable the zero or one encoding is specific to each sample and each variable and does not carry over any distribution information for that variable among the set of samples. In model validation, data arranged by train–test splits do not contain such distribution information that might have been introduced by other pre-processing procedures and the care to apply the whole pre-processing pipeline to the train and test sets is, thus, not necessary.

### 3.2. General Applicability of BinSim

Although in this study we benchmarked BinSim using as examples datasets obtained in two different instruments, with different characteristics in terms of class/group separation, class number, and missing value abundance, and observed quite acceptable performance in clustering and classification methods, we cannot completely generalize the use of BinSim on all types of metabolomics data. A few pitfalls and specific characteristics preclude that generalization.

BinSim uses the contrast of missing to non-missing values in features as a key source of information to be extracted from data. It is predictable that BinSim will degrade the performance of downstream methods if missing-value abundance is either too low or too high. In both cases, there would be few contrasting variables in terms of missing values to allow for good group/class discrimination. In our examples though, even for *YD 6/15*, the dataset with the lowest fraction of missing values, we did not observe such a problem. A systematic study of the effect of BinSim with the overall percentage of missing values falls out of the scope of this work. Our purpose was to compare the effect of BinSim on discriminatory analysis of real datasets and a controlled comparison of the effect of missing value abundance is best carried out with synthetic datasets. However, it is expected that, in ultra-high-resolution metabolomics datasets, missing-value abundance will generally fall within the appropriate range for BinSim to work. In low-resolution metabolomics data, with data matrices constructed from binning with comparably large bins, missing values might be too scarce.

Another concern for the general applicability of BinSim relates to the fact that BinSim transforms with the same coding scheme all the missing values in a data matrix regardless of their type: The distinction of MNAR versus MAR/MCAR values [14] is not made, and, by relying on missing-value occurrence, the performance of downstream methods after BinSim is dependent on the high relative abundance of MNAR values, as these are informative in contrast to MAR/MCAR values. Since the assessment of the type of missing values present in a given sample is usually very hard, it is difficult to establish if the BinSim pre-treatment can, in general, be applied to datasets with a high abundance of MAR/MCAR values. However, the use of two different imputation methods in this study and the results with the dataset *HD* suggest that BinSim may, in fact, be robust to the presence of MAR/MCAR values, if MNAR are still abundant. In comparison studies of different imputation methods [14,25], RF imputation was shown to outperform other imputation methods if MAR/MCAR are abundant. However, if a data matrix contains almost exclusively MNAR values, then imputation methods of the limit-of-detection type, as the 1/2 min imputation method used in this work, may outperform RF imputation [14,25]. Although these conclusions were not established by the effects of imputation method on clustering methods and classifiers, we can compare our results for the non-BinSim pre-treatments with either method of imputation and infer that when performance is higher with RF-imputation than with 1/2 min imputation, then MAR/MCAR values may be present in the dataset. Considering the PLS-DA classifier results, *GDg2*− might be such a case. However, the results with the RF classifier favor 1/2 min imputation in the same example. Furthermore, the results for the *HD* dataset also support the robustness of BinSim to MAR/MCAR values as, in large datasets, the probability of existing missing values of these types increases. Here, for PLS-DA, the performance of classifiers after pre-treatments beginning with RF-imputation was higher than 1/2 min imputation. In these two cases, BinSim did not impair the performance of the classifiers since it was never the worst pre-treatment and the difference to the top pre-treatment was always slight. This may be a sign of the robustness of BinSim to MAR/MCAR values.

A characteristic that does not generalize the use of BinSim in every metabolomics study is what is concluded from the feature importance comparison. After BinSim, RF classifiers “seek” features that occur in very few classes and with sample occurrence that corresponds to the number of samples in those classes. These “biomarker-type” features may not be what a researcher wants to investigate. Instead, features that are known to occur in every class and are considered of interest in a targeted metabolomics study need to be assessed by methods used after intensity-based pre-treatments. In this context however, analysis based on BinSim-treated data may still be considered complementary to other methods and it may be envisaged as “another tool” available to the metabolomics data analyst.

Another concern that may rise during the use of BinSim as a pre-treatment stems from the fact that BinSim does not provide any form of feature selection per se. In this study’s benchmark, feature selection was also not included in any combination of intensity-based pre-treatment steps. This was because feature selection methods are very diverse and there is no obvious general adaptation of the feature selection methods commonly found in metabolomics data analysis to BinSim. Selection of variables relying on feature importance, with the purpose of iteratively improving model performance are independent of pre-treatments and should present no problem if used to complement BinSim. Variable rejection based on an excessive number of missing values can still be applied, but specific thresholds must be adapted taking BinSim into consideration for the reasons discussed above about the dependence of BinSim on missing-value abundance. Most likely, these thresholds must be different from those used in intensity-based pre-treatments, but without a systematic study on the effect of missing-value abundance on BinSim effectiveness, we cannot anticipate any general rule for the application of this form of feature selection. A third type of feature selection, commonly implemented and used as the default in metabolomics data analysis software (in MetaboAnalyst [26], for instance), is the filtering of variables by their variability among the ensemble of samples, where variables with low values of a dispersion statistic are discarded. There is no direct counterpart of this selection after BinSim was already applied. A procedure which could be considered analogous to this type of filtering would be to specify a threshold for Gini impurity of variables: variables with non-missing values distributed by many classes would not be considered as carrying useful occurrence information and should be discarded. An investigation of the effect of this step, to be applied after BinSim in the pipeline, falls out of the scope of this study but may be established as an interesting complement that falls within the rationale behind BinSim.

In this study we performed a form of feature selection: The rejection of variables with a very low number of sample occurrence (one count in the set of all samples) was performed prior to the application of all pre-treatments. This was justified by a reproducibility criterion, as such variables may be considered the result of noise or excessive variability but close to the limit of detection. This form of selection affected all pre-treatments in our study, and it is a procedure that was not applied with the argument of performance improvement in mind. Above all, it had the effect of significantly reducing the number of variables, most of which would not carry any information for further analysis whether in occurrence or intensity. However, a more stringent form of this selection was also employed: In datasets *GDc2*+ and *GDc2−*, variables with a single occurrence in each class (or group) were discarded. The effect of this type of filtering is apparent when *GDg2+* and *GDg2*− are compared with *GDc2*+ and *GDc2−*: All methods improved performance, especially after the BinSim transformation. This was expected, since it improves the likelihood of variables becoming characteristic of classes and effectively “spikes” their occurrence in those classes and samples of the same class are artificially made more similar to each other, something that benefits methods used on BinSim data. This form of filtering, that is, justified by performance improvement arguments, should be used with care, since it may lead to a form of data leakage: When using internal validation methods, “test samples” used to validate the models were made artificially closer to the training samples. The use of this type of filter in training data in a discriminatory analysis context requires the model to be validated by an external metric so that testing samples are always treated as truly “unseen samples”.

BinSim, as a pre-treatment method, might prevent some types of analysis that rely heavily on intensity-based data. Univariate methods are no longer possible and value predictions from regression methods might also require data that are not binarized. We showed that BinSim works well for clustering and classification, which are common in metabolomics data analysis but its effect on the performance of regression methods needs to be established by a systematic comparison, conceptually similar to the one performed in this study.

As proposed in this study, BinSim behaves as a binary encoding of feature occurrence in the data matrix and is akin to the binary encoding of categorical variables. However, issues resulting from the introduction of such encodings in the explanatory data matrix have been pointed out for some situations. For instance, when the explanatory data matrix combines variables of different measurement scales, such as categorical and interval or ratio scales, PLS based methods, including PLS-DA, must be adapted to ensure optimal scaling [27]. To avoid these problems, BinSim should not be used with such heterogenous data for the development of a PLS-DA model, even in the case where BinSim is applied to variables of only one type. In this study, in all the benchmark examples, BinSim was applied to the whole data matrix that contained only metric (interval or ratio scale) data.

## 4. Materials and Methods

### 4.1. Datasets

The main goal of this study was the comparison of the effects of Binary Simplification and traditional pre-treatments of the data matrix in downstream metabolomics data analysis. For that purpose, we constructed eight data matrices from experimental data which encompass different levels in terms of number of features and abundance of missing values. Seven of the data matrices (datasets) were based on three metabolomics datasets obtained from two different Fourier transform ion-cyclotron resonance mass spectrometry (FT-ICR-MS) instruments, with different mass accuracies and resolutions. The eighth dataset was obtained in an Orbitrap instrument coupled with a hydrophilic interaction chromatography (HILIC).

“Grapevine datasets” were constructed from already published and openly available data [28] related to a study of the metabolome differences observed in *Vitis* varieties that are susceptible or resistant to oomycete/fungal infections [29]. Detailed description of metabolomics data acquisition is described in the article and in the data deposition site [28,29]. Briefly, leaf samples of different *Vitis* plants were analyzed by direct infusion in a 7T-Apex FT-ICR-MS, by electrospray ionization in positive (ESI+) and negative (ESI−) modes [29]. The data consist of 3 biological replicates of 11 different grapevine genotypes [28], and data from the positive and negative ionization modes were treated independently. The 33 total samples were aligned together by a peak-based method using the metabolinks Python package [30] at 1 ppm *m*/*z* distance tolerance, generating a data matrix with 5821 peaks in the negative mode and a data matrix with 30,660 peaks in the positive mode. From these datasets, several matrices were constructed by performing different levels of a variable selection related to spectral feature reproducibility (Table 1): matrices *GDg2+* and *GDg2*− were generated after retaining only features that occur (globally) at least twice in all 33 samples, for each of the acquisition modes; matrices *GDc2*+ and *GDc2−* were generated after retaining only features that occur at least twice in the three replicates of at least one *Vitis* variety (class), for each of the acquisition modes. In the assessment of the performance of supervised methods, an additional dataset was created, suitable for fitting two-class classifiers. This is just dataset *GDc2−* with class labels “vinifera” and “wild”, corresponding to grapevine varieties of *Vitis vinifera* and to wild *Vitis* (non-vinifera) species, respectively. This dataset is referred to as *GD types*.

“Yeast datasets” were built from data obtained from direct infusion analysis by electrospray ionization in a 7T-Solarix XR FT-ICR-MS, operating in positive (ESI+) ionization mode [31,32] and available in the *figshare* public repository [33]. The data consist of 3 replicates of 5 different strains of *Saccharomyces cerevisiae*: the reference strain BY4741 (represented as wild type, WT), 3 single-gene deletion mutants of this strain, related to methylglyoxal metabolism—ΔGLO1, ΔGLO2, ΔGRE3, and the single-gene-deletion control ΔENO1. The raw data from the 15 samples were aligned using the MetaboScape 4.0 software (Brüker Daltonics, Bremen, Germany) using the T-ReX (Time aligned Region complete eXtraction) algorithm with the following parameters: *m*/*z* delta = 1.10, Intensity Threshold = 0.00, Maximum Charge = +1. From the resulting “bucket table”, two datasets were constructed by selecting features based on its reproducibility with different thresholds: In dataset *YD 2/15*, features that occurred in at least 2 samples were retained, and in dataset *YD 6/15*, features that occurred in at least 6 samples were retained.

“Human dataset”, here referred to as *HD*, was built from data obtained from a study of the preoperative metabolic signatures associated with prostate cancer recurrence versus remission after radical prostatectomy [34]. These data are available at the NIH Common Fund’s National Metabolomics Data Repository (NMDR) website, the Metabolomics Workbench, project ID PR000724 (study ID: ST001082). Detailed metabolomics data acquisition is described in the study and in the data deposition site. Briefly, blood serum of 80 patients, collected before radical prostatectomy, was analyzed by Hydrophilic Interaction Chromatography (HILIC)-MS. Chromatography was performed with a Waters XBridge BEH HILIC column (2.1 × 75 mm, 2.5 μm particle size) in a Thermo Dionex Ultimate 3000 and MS analysis was performed in a Thermo Q Exactive HF hybrid Orbitrap operating in positive electrospray ionization (ESI+) mode [34]. The available data from Metabolomics Workbench consist of 135 MS spectra samples from patients in prostate cancer remission (‘No Recurrence’ class) and 114 MS spectra samples from patients with recurrence of prostate cancer (‘Recurrence’ class). Raw data were aligned and peak picked using the Progenesis QI software package (Nonlinear Dynamics, Waters Corp., Milford, MA, USA), [34]. The average of five blank samples were subtracted from the dataset with emerging negative values coded as 0. From this data, features that occurred in at least 2 samples were retained.

A general characterization of all eight datasets (*GDg2*−, *GDg2+*, *GDc2−*, *GDc2*+, *YD 2/15*, *YD 6/15*, *GD types*, and *HD*) is found in Table 1. Before the data pre-treatments compared in this work, a preliminary assessment of the extent of class/group’s proximity, and consequent degree of difficulty for clustering and classification methods, was performed. For this purpose, Principal Component Analysis scores plots of these datasets were obtained and shown in Figure S1, with indication of each sample class. For this preliminary analysis, data were pre-treated with missing value imputation by half of the global minimum of non-missing values and auto-scaling.

### 4.2. Data Pre-Treatments

Seven data pre-treatment procedures were applied independently to all datasets: Binary Simplification, showcased in this work, and, for comparison, combinations of some of the most established intensity-based methods. These methods were chosen to represent the different stages of pre-treatment in metabolomics data analysis: imputation of missing values, normalization, transformation, and centering/scaling.

Except for BinSim, two missing-value imputation procedures were applied before any treatment:

- Half min: missing values were replaced with half of the minimum intensity value present in the whole data matrix. This is a limit of detection type of missing value imputation commonly applied in metabolomics data analysis.
- RF imputation: Random Forest missing-value imputation [18], where a Random Forest regression method is used to estimate missing values from a number of similar features. The number of trees in RF was set to 50 and the number of similar features to 100.

Each missing-value imputation procedure was then followed by combinations of three pre-treatment methods:

- P: Pareto scaling was applied to either 1/2 min- or RF-imputed data.
- NP: samples were normalized by the reference feature (leucine enkephalin, for ESI+ data, *m*/*z* 556.276575; for ESI− data, *m*/*z* 554.262022). Dataset *HD* was normalized by Probabilistic Quotient Normalization [19]. Pareto scaling was then applied. The two steps were applied to either 1/2 min or RF-imputed data.
- NGP: samples were normalized as in NP and then transformed by generalized logarithmic transformation and scaled by Pareto scaling. The three steps were applied to either 1/2 min- or RF-imputed data.

The Binary Simplification procedure (BinSim) considers the occurrence of spectral features to construct a binary data matrix encoding feature presence (with intensity values) as 1 and absence (missing values) as 0. No further transformation was applied.

In total, seven different combinations were applied to each dataset: P, NP, and NGP applied to either 1/2 min- or RF-imputed data and BinSim.

### 4.3. Assessment of the Effect of Pre-Treatments

BinSim was compared with the other pre-treatments regarding the effect on the performance of selected clustering and classification methods representing the two general types of unsupervised and supervised methods used in metabolomics data analysis. For clustering, performance was assessed by the ability of the methods to correctly cluster replicates of the same type, species or strain of the organisms that contribute to the different datasets. These groups, defined by the replicates, are the “ground truth” of correct clusters allowing the use of ground-truth related metrics of clustering performance. Clustering was applied after each pre-treatment for datasets *GDg2+*, *GDg2*−, *GDc2*+, *GDc2*+, *YD 2/15*, and *YD 6/15*. For classifiers, these replicates of the same type, species, or strain also define the target classes to be predicted, except for the *GD types* dataset where two classes were defined as described above, considering wild *Vitis* plants versus *Vitis vinifera* plants and the *HD* dataset where the two classes represent patients with cancer recurrence versus cancer remission (binary classifier problems). For classifiers, predictive accuracy was the main performance goal to be assessed. Clustering methods assessed were Agglomerative Hierarchical Clustering (HCA) and K-means Clustering, and classifiers methods were Random Forest (RF) and Projection in Latent Structures Discriminant Analysis (PLS-DA).

### 4.4. Clustering Methods

Agglomerative Hierarchical Clustering Analysis with UPGMA (average) linkage method was performed on each dataset. Euclidean distance was used for the datasets treated with intensity-based pre-treatments, whereas, for the BinSim-treated datasets, three dissimilarity metrics were chosen due to their binary natures: Jaccard and Yule dissimilarities and Hamming distance.

For two samples, S1 and S2, if $n_{11}\;$is the number of features present in both samples, $n_{00}\;$is the number of features absent from both samples, $n_{10}\;$is the number of features present in sample 1 but not in sample 2, and $n_{01}$ is the number of features present in sample 2 but not in sample 1; these dissimilarity metrics are defined as follows:

- Jaccard dissimilarity [35]:
  (1) $$D_{\mathrm{Jaccard}}\;(S1,\;S2)=1-\frac{n_{11}}{n_{11}+n_{10}+n_{01}}\;$$
- Yule dissimilarity [36]:
  (2) $$D_{\mathrm{Yule}}\;(S1,\;S2)=\frac{2\times {\;n}_{10}\times {\;n}_{01}}{n_{11}\times {\;n}_{00}+{\;n}_{10}\times {\;n}_{01}}\;$$
- Hamming distance [37]:
  (3) $$D_{\mathrm{Hamming}}\;(S1,\;S2)=\frac{n_{10}+{\;n}_{01}}{n_{11}+{\;n}_{00}+{\;n}_{10}+{\;n}_{01}}$$

Clustering performance was expressed by three metrics. The “correct clustering” percentage is defined as the percentage of the groups whose samples all clustered together before any other clustering with other samples or already formed clusters in the agglomerative procedure. The global “Discrimination Distance” (DD) is defined as the average of “group discrimination distance”. For each group, the discrimination distance is 0 if the group is not “correctly clustered” (in the sense used for the correct clustering metric) or it is the distance between the node that includes all the samples of the group and the next closest node (including those samples) in the agglomerative procedure, normalized by the maximum distance of any pair of nodes in the final resulting clustering. The “correct first cluster” percentage is defined as the percentage of samples whose first clustering was only with a sample(s) from its group. The similarity between the dendrograms obtained for the same dataset with different pre-treatments was evaluated by the cophenetic correlation coefficient [20] and the Baker’s gamma correlation coefficient [21]. The computation of these coefficients was adapted from the R package dendextend version 1.15.1 [38].

K-means Clustering Analysis was applied per dataset and treatment parameterized with a cluster number equal to the total number of classes/groups (11 in *GD* and 5 in *YD*) and using Euclidian distance (including for the BinSim-treated datasets). As K-means Clustering results can vary with the randomness of initial centroid assignments [39], the algorithm was repeated 15 times and retaining the result with the least inertia. Clustering performance was expressed by three metrics: as in HCA, discrimination distance and correct clustering percentage (group-based metrics) were computed but with a slight redefinition of the concept of “correct clustering”. In this case, the distances are measured between cluster centroids and a clustering is correct if it contains all and only the samples of a single group (total homogeneity and total completeness). This is a stricter condition that the one imposed in the HCA, so a lower percentage of correct clustering is expected. The third metric was the Rand Index (a sample-based metric), a measure defined by the proportion of sample pairs which are correctly clustered or correctly not clustered, adjusted for the expected percentage of samples which would be in those situations randomly.

### 4.5. Classifiers

The classifiers chosen to use as a comparison test between the different pre-treatments were Random Forests and PLS-DA. Except for the *GD types* problem, where the 2 classes have 15 and 18 samples each and the *HD* problem where the 2 classes have 114 and 135 each, the other datasets have a low number of replicates and, by the definition of the classification target, a low number of samples in each class. Therefore, validation of the models was done by internal stratified 3-fold cross-validation [22] and 5-fold stratified cross-validation in the *GD types* and *HD* problems. The performance of the models was judged based on their average prediction accuracy in cross-validation. Since the random splits of the samples in each fold can affect the results (even if slightly), especially with low sample size in each class, this process was iterated 20 times and the mean accuracy of all repetitions was taken as a global metric for the cross-validation evaluation.

The number of trees used for the Random Forest classifiers was optimized to 100. Other parameters were left as the default values used in the scikit-learn RandomForestClassifier object constructor. For each model, the Gini Importance of each feature was calculated [23]. The top 2% of Gini Importance features were retained and their relevant characteristics were evaluated, namely, the number of samples and classes where they occur.

For the two-class *GD types* classification problem, a ROC curve for each of the three pre-treatments starting with 1/2 min imputation and for BinSim was also computed.

Projection in Latent Structures Discriminant Analysis (PLS-DA) classifiers were built for each dataset and each pre-treatment using the PLS2—NIPALS algorithm implemented in PLSRegression module of scikit-learn [40]. The default parameters in scikit-learn were used, except the scaling of the samples, not performed since data were already pre-treated. The number of components for the PLS-DA models were chosen to minimize the predictive residual sum of squares (maximize Q^2^) computed from cross-validation. Moreover, 11 components were chosen for all *GD* datasets, 6 components for the *YD* datasets and *GD types*, and 15 components for the *HD* dataset. Class membership was encoded by the one-hot encoding method, and the prediction decision samples were assigned to the class corresponding to the maximum value in y_pred_ of the PLS output. In the *GD types* and *HD* problems, class membership was encoded as 0 or 1, with a 0.5 threshold for decision. As it was conducted for the Random Forest, the top 2% of the features considered as most important were taken to build the models, and their sample and class occurrence was calculated. Here, the Variable Importance in Projection (VIP) was used to estimate the importance of each feature to build each model [24].

For both RF and PLS-DA methods, permutation tests (with 500 iterations each) were performed to further assess if the models were significant.

### 4.6. Implementation

Data transformations, clustering methods, and classifiers were implemented in the Python language version 3.8.5 using pandas version 1.2.4 [41] and scikit-learn version 0.24.2 [40] packages using the Python module metabolinks version 0.71 [30]. The code that supports this study is available at the repository https://github.com/aeferreira/binsim_paper since 16 November 2021.

## 5. Conclusions

The Binary Simplification (BinSim) pre-treatment was specifically created with metabolomics’ data analysis in mind as a simpler and reliable alternative to traditional pre-treatments. It focuses on the presence or absence of features instead of the intensity-based nature of the other pre-treatments. The occurrence of features leads to the construction of binary data matrices encoding feature presence as 1 and absence as 0. Despite this binarization and aggressive truncation of intensity information, the performance of both clustering and classification methods was not compromised. HCA, K-means Clustering, PLS-DA, and RF did perform consistently as well or, often, better with BinSim-transformed data than with data subjected to combinations of other intensity-based pre-treatments. Furthermore, the general trends observed in the comparison of performances were also consistent between the different methods.

With RF models, the characteristics of important features are distinctive: with BinSim-treated data, classifiers tend to attribute higher importance scores to features with sample occurrences characteristic of indicators of one or very few classes/groups, while, with data subjected to intensity-based pre-treatments, the distribution of sample occurrence of important features tends to be more uniform or less related to class occurrence. After BinSim, classifiers score features according to a “different perspective” on data.

BinSim, as pre-treatment, substitutes several steps in a metabolomics data analysis pipeline, in particular, missing-value imputation, normalization, and scaling. The pre-treatment stage of data analysis becomes straightforward, as the need for choices of methods and method parameter vanishes.

While not generalizable to all scenarios in metabolomics data analysis, BinSim is a viable alternative to intensity-based pre-treatments for high-resolution, high-mass-accuracy untargeted data, where missing-value occurrence carries a significant information content. Since metabolomics studies that generate data with these characteristics are currently becoming common, we propose the adoption of BinSim as pre-treatment step in metabolomics data-analysis.

## Figures and Tables

**Table 1:** General characteristics of the benchmark datasets: Detailed description of the datasets is presented in Materials and Methods section.

Dataset	Samples	Features	Features/Sample (Range)	Classes	Samples/Class	Missing Values (%)
*GDg2*−	33	3629	658 (367–1002)	11	3	81.86
*GDg2*+	33	7026	1164 (355–2141)	11	3	83.43
*GDc2*−	33	3026	547 (338–919)	11	3	81.91
*GDc2*+	33	4565	824 (215–1670)	11	3	81.94
*GD types*	33	3026	547 (338–919)	2	15 *V. vinifera*,	81.91
					18 wild *Vitis*	
*YD 2/15*	15	1973	685 (584–757)	5	3	65.27
*YD 6/15*	15	606	468 (383–514)	5	3	22.76
*HD*	249	12,869	7936 (7057–8475)	2	114 “Recurrence”, 135 “no Recurrence”	38.33

## Data Availability

Grapevine MS data used in this work are openly available in figshare data repository with the identifier https://doi.org/10.6084/m9.figshare.12357314, accessed on (15 August 2021). Yeast MS data used in this work are openly available in figshare data repository with the identifier https://doi.org/10.6084/m9.figshare.15173559, accessed on (1 September 2021). Human MS data used in this work can be accessed at Metabolomics Workbench directly via its project, https://doi.org/10.21228/M83D5V, accessed on (15 October 2021).

## Supplementary Materials

The following are available online at https://www.mdpi.com/article/10.3390/metabo11110788/s1, Figure S1: Principal Component Analysis scores plots for the benchmark datasets, Figure S2: Dendrograms resulting from the application of HCA to dataset *GDc2*+ with different pre-treatments, Figure S3: Receiver Operating Characteristic curves for Random Forest models fitted to the *GD types* dataset, Figure S4: Permutation tests for some of the Random Forest models, Table S1: Effect of pre-treatments on clustering performance of HCA, Table S2: Effect of pre-treatments on clustering performance of K-means, Table S3: Permutation tests for the supervised methods.
