# Supplementary material for: Binary Simplification as an Effective Tool in Metabolomics Data Analysis
Source: Metabolites. 2021 Nov 18;11(11):788. doi: 10.3390/metabo11110788 (PMC8621519; doi:10.3390/metabo11110788)
Supplement: Supplementary file 1 [file metabolites-11-00788-s001.zip › supplementary_figures.pdf]

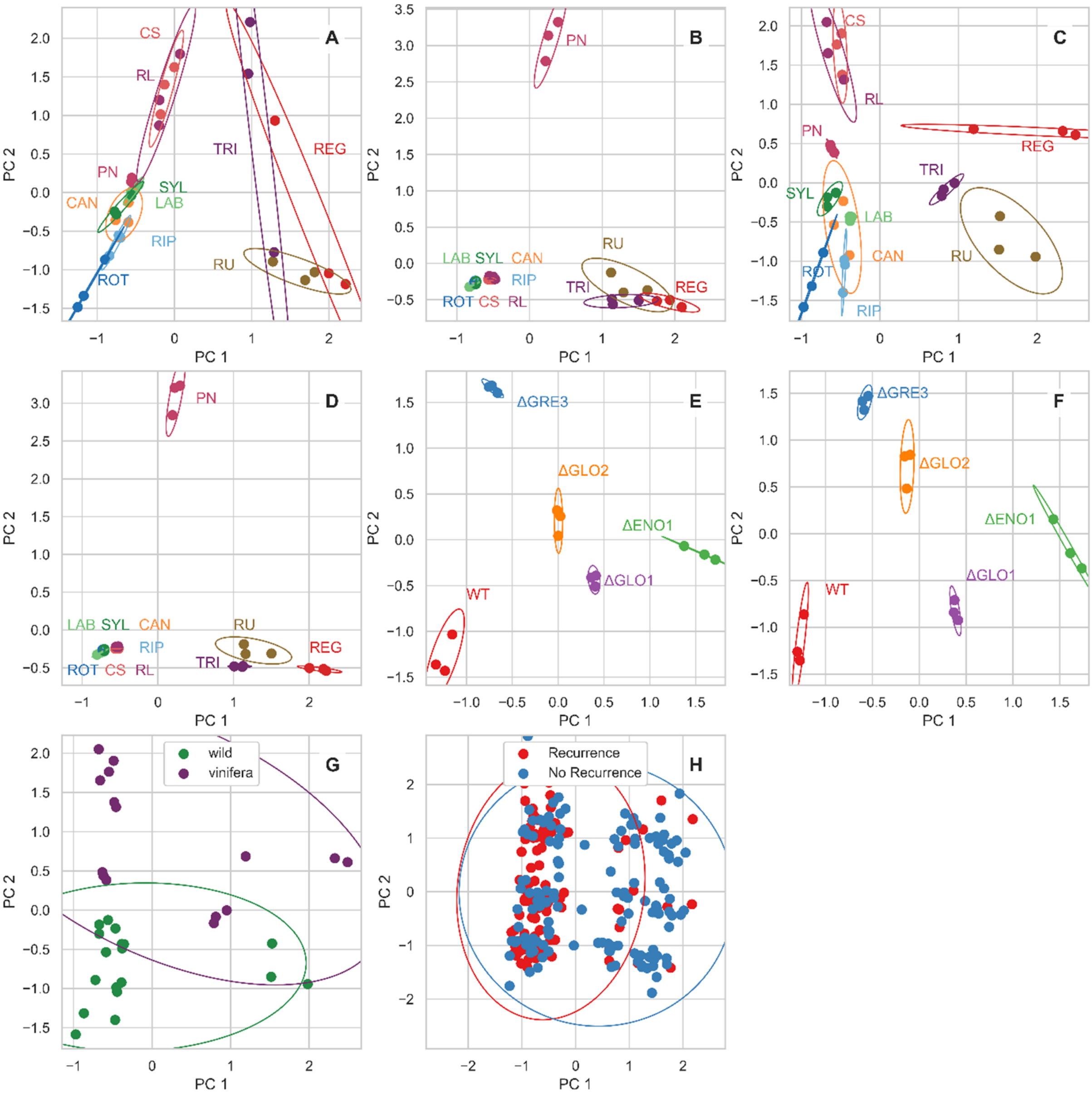

Figure S1. Principal Component Analysis scores plots for the benchmark datasets. (**A**) *GDg2-*; (**B**) *GDg2+*; (**C**) *GDc2-*; (**D**) *GDc2+*; (**E**) *YD 2/15* (**F**) *YD 6/15* (**G**) *GD types*; (**H**) *HD*; All datasets were pre-treated with constant missing-value imputation equal to ½ of the non-missing global minimum in the data matrix and auto-scaling. Labels identify replicates of each class defined for the data. Ellipses are 95% confidence ellipses for each class.

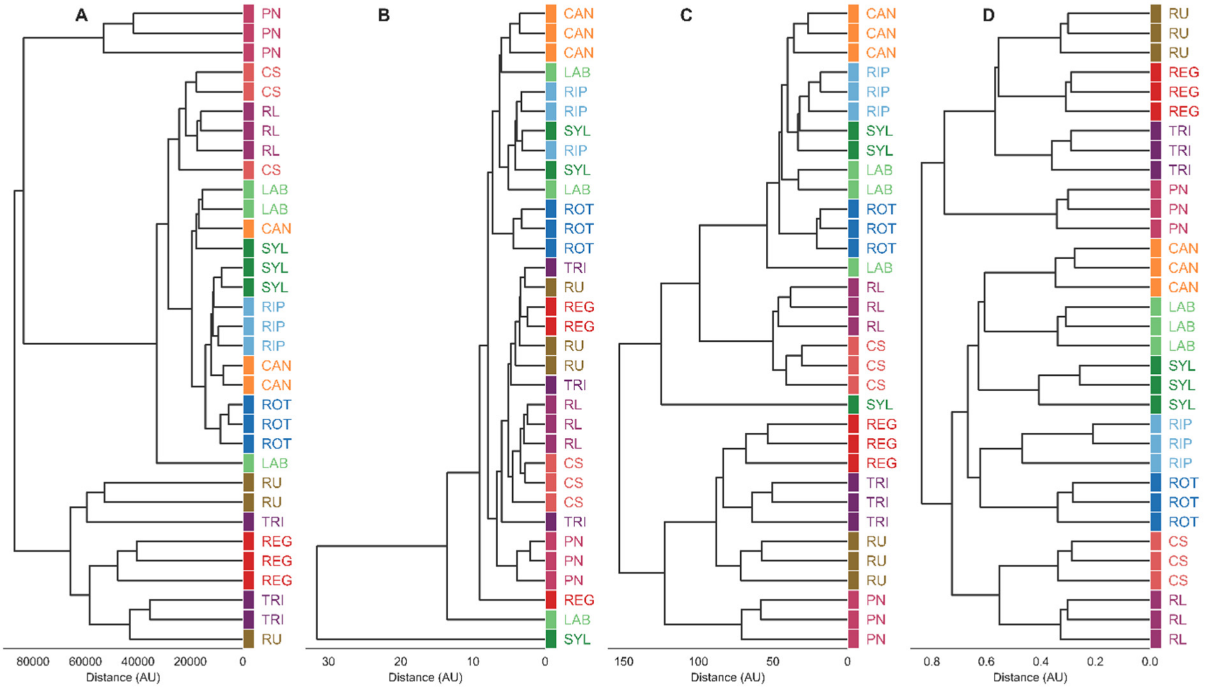

Figure S2. Dendrograms resulting from the application of HCA to dataset *GDc2+* with different pre-treatments. (**A**) Pareto scaling only (P), Euclidean distance; (**B**) normalization by reference feature and Pareto scaling (NP), Euclidean distance; (**C**) normalization by reference feature and *g-log* transformation and Pareto scaling (NGP), Euclidean distance; (**D**) Binary simplification only (BinSim), Jaccard distance. ½ min missing-value imputation was used in these examples. Average linkage for HCA was used in all cases.

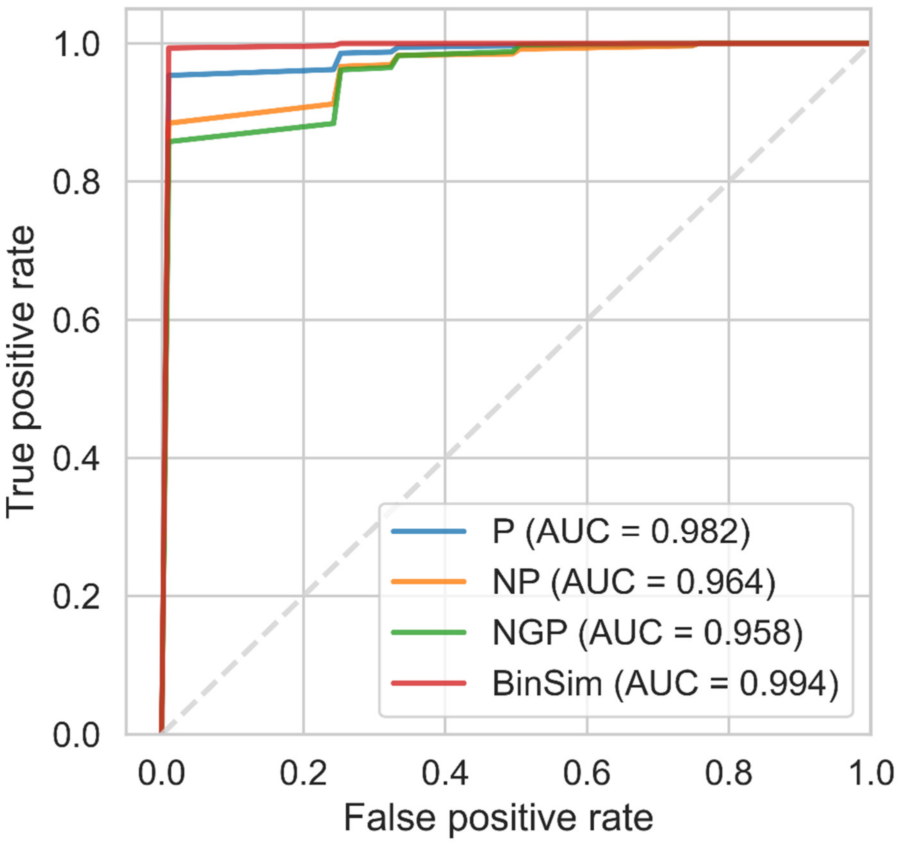

Figure S3. Receiver Operating Characteristic curves for Random Forest models fitted to the *GD types* dataset. AUC: Area Under the Curve. Data pre-treatments: Pareto scaling only (P); normalization by reference feature and Pareto scaling (NP); normalization by reference feature and glog transformation and Pareto scaling (NGP); Binary simplification only (BinSim). Except for BinSim, ½ min missing-value imputation was used before the other pre-treatments.

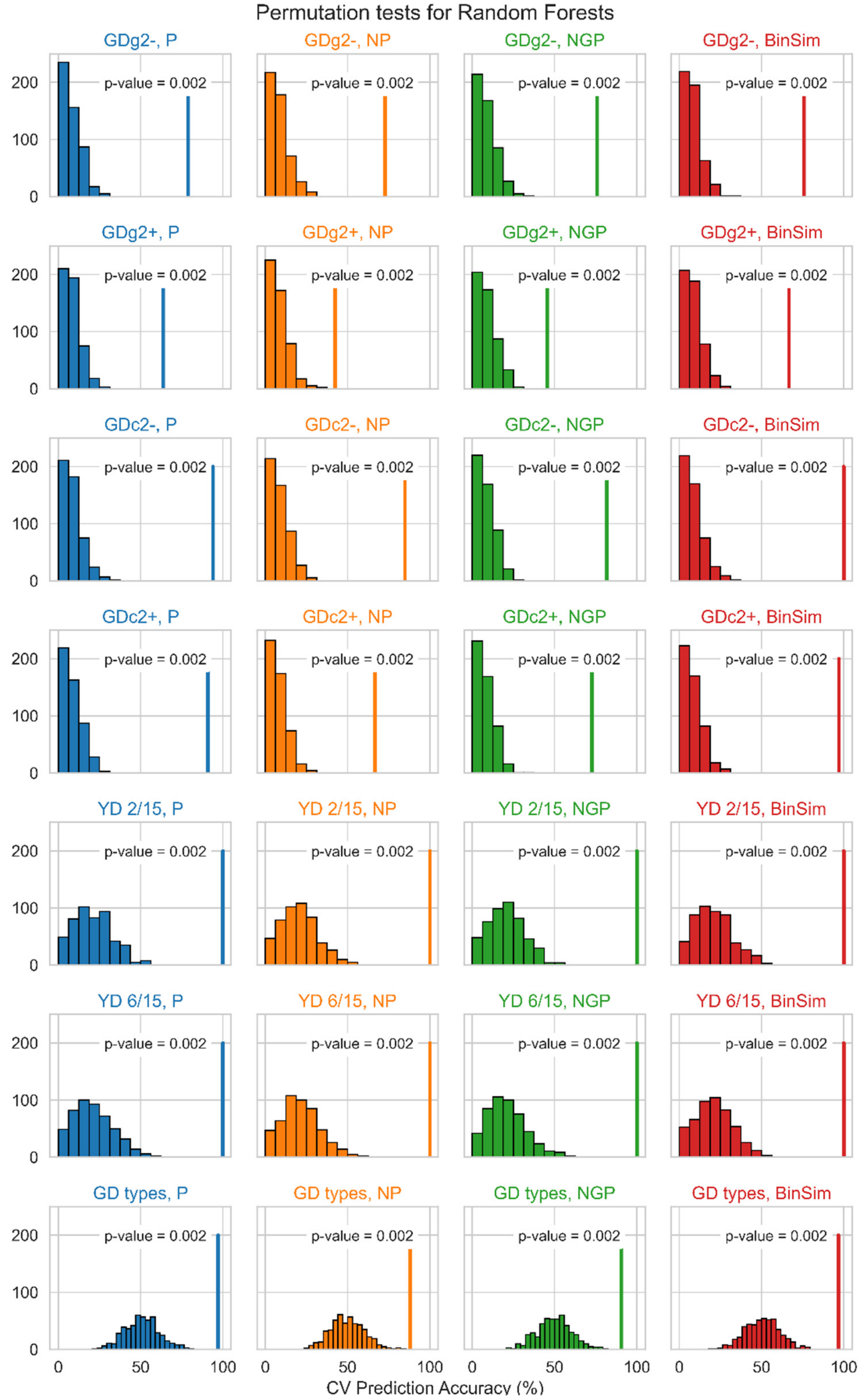

Figure S4. Permutation tests for some of the Random Forest models. The distribution of prediction accuracy of 500 permutations of sample labels is shown. Vertical lines indicate the accuracy of the model without label permutations. Except for BinSim, ½ min missing-value imputation was applied. Data pre-treatments: Pareto scaling only (P); normalization and Pareto scaling (NP); normalization and Pareto scaling and glog transformation (NGP); Binary simplification (BinSim). *p*-values for all the models are indicated in Table S3.
